# Abnormal interhemispheric functional connectivity in patients with strabismic amblyopia: a resting-state fMRI study using voxel-mirrored homotopic connectivity

**DOI:** 10.1186/s12886-021-02015-0

**Published:** 2021-06-09

**Authors:** Shuang Zhang, Gui-Ping Gao, Wen-Qing Shi, Biao Li, Qi Lin, Hui-Ye Shu, Yi Shao

**Affiliations:** Department of Ophthalmology, The First Affiliated Hospital of Nanchang University, Nanchang, Jiangxi China

**Keywords:** Strabismic amblyopia, fMRI, VMHC, Functional connectivity

## Abstract

**Background:**

Previous studies have demonstrated that strabismus amblyopia can result in markedly brain function alterations. However, the differences in spontaneous brain activities of strabismus amblyopia (SA) patients still remain unclear. Therefore, the current study intended to employthe voxel-mirrored homotopic connectivity (VMHC) method to investigate the intrinsic brain activity changes in SA patients.

**Purpose:**

To investigate the changes in cerebral hemispheric functional connections in patients with SA and their relationship with clinical manifestations using the VMHC method.

**Material and methods:**

In the present study, a total of 17 patients with SA (eight males and nine females) and 17 age- and weight-matched healthy control (HC) groups were enrolled. Based on the VMHC method, all subjects were examined by functional magnetic resonance imaging. The functional interaction between cerebral hemispheres was directly evaluated. The Pearson’s correlation test was used to analyze the clinical features of patients with SA. In addition, their mean VMHC signal values and the receiver operating characteristic curve were used to distinguish patients with SA and HC groups.

**Results:**

Compared with HC group, patients with SA had higher VMHC values in bilateral cingulum ant, caudate, hippocampus, and cerebellum crus 1. Moreover, the VMHC values of some regions were positively correlated with some clinical manifestations. In addition, receiver operating characteristic curves presented higher diagnostic value in these areas.

**Conclusion:**

SA subjects showed abnormal brain interhemispheric functional connectivity in visual pathways, which might give some instructive information for understanding the neurological mechanisms of SA patients.

## Introduction

**Fig. 1:**
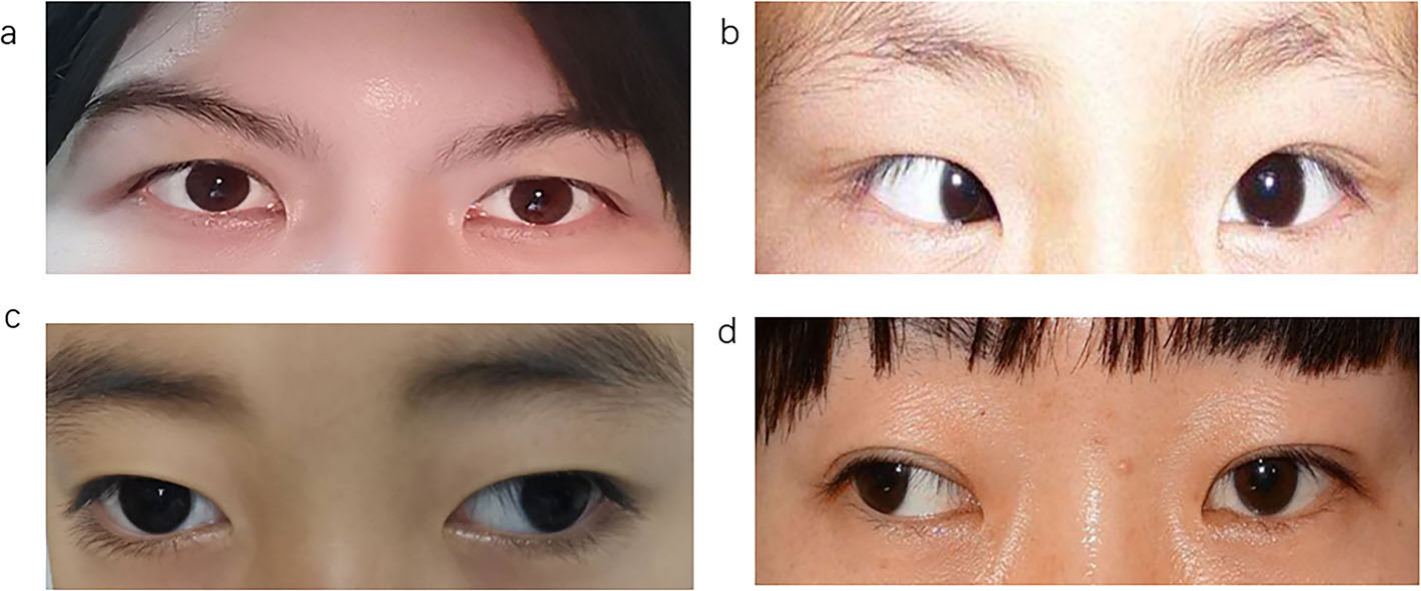
The eyes of the esotropia patient and the healthy control. Notes: **a** Represents the eye position of healthy people. **b** Represents the eye position of esotropia. **c** and **d** denote the eyes position of exotropia

Amblyopia is the decline of monocular or binocular best-corrected visual acuity caused by abnormal visual experience during visual development [1, 2]. Eye examination does not indicate the presence of organic lesions and the prevalence of amblyopia in the general population is 2–4% [3]. The normal development of visual cortical neurons in the critical period of maturity requires normal visual stimulation, during which any abnormal visual experience can result in amblyopia. The most common types of amblyopia refers to strabismus, anisometropia, or both of the two [4]. Strabismus is an eye movement disorder that can impair stereoscopic vision [5]. SA is a syndrome usually caused by uncorrected misalignment of the visual axis. It is described as defects in stereoscopic depth perception and visual sensitivity [6], as well as damage in spatial positioning, gaze, eyeball movement and adjustment [7]. Figure 1 presents the eye of patients undergoing SA and healthy control.

Amblyopia is generally regarded as a neurodevelopmental visual disorder, and its neural basis has been extensively investigated. However, the exact mechanism involved in such condition is not completely clear [8]. Recently, some non-invasive neuroimaging examinations have found that amblyopia is limited to primary visual cortex (V1) functional and structural defects, and cascades to the higher visual cortex and related brain areas of abnormalities [9], indicating the existence of visual processing defects [8]. Previous studies have already demonstrated that blood oxygen level-dependent signal in visual cortex and cerebral blood flow altered in SA patients. Specific area of the brain is activated such as the lateral geniculate nucleus of amblyopia [10]. In addition, these areas showed activity in various strabismus studies. Therefore, impairment of SA function leads to abnormalities in the spatial characteristics of cortical neurons and involves multiple brain regions, resulting in a combination of multi-directional and multi-level damage to visual nerves [11]. The study conducted by Rossion et al. [12] revealed that functional impairment of SA is associated with abnormalities in the spatial characteristics of cortical neurons and involves multiple brain regions. The difference can be found by the sensitivity of the two visual hemispheres to various visual information. The synchronization between the cerebral hemispheres is closely associated with the visual experience. The lack of interaction between the hemispheres may lead to the visual impairment of amblyopia. Additionally, previous studies have also found that the synchronization between the cerebral hemispheres is closely related to the visual experience [13]. Resting-state fMRI can detect focal brain activation and interhemispheric coordination [14]. Voxel-mirrored homotopic connectivity (VMHC), local consistency (regional homogeneity) and amplitude of low-frequency fluctuation (ALFF) can be obtained from resting-state fMRI. Starting from the resting brain symmetrical voxel, VMHC is an index which can be used to measure the synchronization of two hemispheres. The intensity of the functional connection between a voxel and its corresponding voxel in the contralateral hemisphere is calculated. The method of abnormal connection of multiple symmetrical regions in the two hemispheres can reflect the coordination of signal activity in the left and right hemispheres. The difference of brain connection between patients with SA and HC group can be compared by VMHC method, which can benefit us in understanding the neuroimaging mechanism of cerebral hemispheric synergy disorder in patients with SA [15, 16]. Due to its non-radioactive, non-invasive, and accurate location, the VMHC method has been used in diseases such as acute open eye injury [17], monocular blindness [18], primary open-angle glaucoma [19] and post-herpetic neuralgia [20]. In the present study, we employed the VMHC method to evaluate the changes in interhemispheric FC in patients with SA under different resting states. This approach is conductive to revealing the neural variation of ocular diseases and exploring the spatial anatomy and function of the brain of patients with SA [21, 22].

## Material and methods

### Participants

A total of 17 patients with SA (eight males and nine females) treated in the First Affiliated Hospital of Nanchang University (Nanchang, China) were included in the current study. The inclusion criteria included: 1) recruitment from the ophthalmology department of our hospital according to the criteria of the “amblyopia diagnosis expert consensus” (2011) [23]; 2) each subject had undergone a comprehensive ophthalmological examination, including assessment of visual acuity, ciliary paralysis optometry, stereoscopic acuity, intraocular pressure, eye movement, and fundus examination; and 3) the absence of other ophthalmopathy (e.g., glaucoma, optic neuritis, corneal ulcer, monocular blindness, etc). The exclusion criteria were presented as following: 1) a history of ophthalmic surgery and other eye diseases (e.g., infection, inflammation, and local ischemic disease); 2) mental disorder (e.g., depression, paranoia); 3) cardiovascular disease (e.g., hypertension, heart failure, atherosclerosis); 4) brain disease; and 5) addiction to drugs or alcohol. In addition, totally seventeen HCs (eight males and nine females) were enrolled. Their age, sex, and education level were similar to those of the SA group. The inclusion criteria were: 1) normal brain parenchyma; 2) no history of ophthalmological disease; 3) absence of symptoms and signs of neurological diseases and brain parenchyma malformations; 4) no mental illness (e.g., depression and delusional disorder); and 5) no history of drug or alcohol addiction. The current study was approved by the research ethics committee of the First Affiliated Hospital of Nanchang University and the protocol was in consistence with the tenets of the Declaration of Helsinki. In addition, every patient involved in this study had been informed of the whole study design and signed the informed consent.

### MRI data collection

The MRI scan was performed using the 3.0-T eight-channel head coil system (Siemens, Munich, Germany). Firstly, the whole brain uses a three-dimensional gradient recall sequence to obtain high-resolution T1-weighted images. The functional data were collected using a 3D spoiled gradient-recalled echo sequence with the following parameters: 176 structural images (repetition time = 1900 ms, echo time = 2.26 ms, thickness = 1.0 mm, gap = 0.5 mm, acquisition matrix = 256 × 256, field of view = 250 × 250 mm and flip angle = 9°) and 240 functional images (repetition time = 2000 ms, echo time = 30 ms, thickness = 4.0 mm, gap = 1.2 mm, acquisition matrix = 64 × 64 field of view = 220 × 220 mm and flip angle = 90°) wereobtained. The total scanning time was approximately 20 min. Cotton balls were inserted in the ears of the subject and the subject’s head was fixed using a sponge foam pad with the aim to reduce noise interference and limit head movement, respectively. The subjects were requested to lay quietly with their eyes closed, breathe normally as well as avoid movement and any mental activities as much as possible.

### fMRI data preprocessing

After obtaining the functional images, the MRIcro software (www.MRIcro.com) was employed to process the functional data. The functional data were analyzed by resting functional magnetic resonance imaging advanced edition (DPARSFA 4.0; http://rfmri.org/DPARSFA) and statistical parameter mapping (SPM12) based on MATLAB2010a (Math works, Natick, MA and USA). In addition, a series of corrections were subsequently performed. (1) the first 10 volumes were discarded as the signal instability caused by incomplete T1 relaxation at the beginning of data acquisition. (2) Time correction: the time difference was corrected during scanning to guarantee that the time acquired by all voxels in a time point was consistent theoretically. (3) Head motion correction: the maximum displacement of x, y, or z was < 1.5 mm and the rotation was < 1.5 mm during the whole fMRI scanning process so as to correct the slight head movement of the subjects between the time points during the scanning process. (4) Spatial standardization: the standard echoplanar image template was employed to standardize the functional images in reaching the Montreal Neurological Institute (MNI) space criteria in order to overcome the difference between the brain structure of different subjects. (5) Spatial smoothing: smoothing can reduce the registration error and increase the normality of the data for statistic. 4 mm × 4 mm × 4 mm full width at half maximum (FWHM) smoothing was used to satisfy the spatial standard of the Montreal Neurological Research Institute. (6) De-linear drift: the effect of the increasing temperature of the machine or fatigue caused by long-term scanning of the subjects were removed. (7) Regression of head movement parameters, white matter, and cerebrospinal fluid signals was performed to reduce the influence of head movement and other signals. (8) Low-frequency filtering: the 0.01–0.08 Hz band was adopted for low-frequency filtering.

### VMHC analysis

Before conducting VMHC statistical analysis, the image is standardized to a symmetrical template. (1) All gray matter images normalized to MNI space were averaged to produce an average image. (2) The present study averaged the resulting average image with the left and right mirrored images, and obtained a symmetrical template for VMHC statistical analysis. (3) The preprocessed gray matter image of each person is registered to the symmetrical template by nonlinear transformation. Then, VMHC analysis is carried out using REST software package (http://www.restfmri.net/forum/REST). The time series of each voxel in the whole cerebral hemisphere that has been preprocessed and registered to the standard Montreal (MNI) space is extracted. In the meanwhile, the Pearson correlation coefficient between each symmetrical voxel in the left and right position in the brain is calculated, which is VMHC value. The relevant result map is converted into a Z-value map for double-sample t-test analysis by Fisher Z conversion.

### Statistical analysis

SA patients’ clinical parameters were analyzed using independent sample t-tests between the two groups by the SPSS 22.0 (SPSS Inc., Chicago, IL, USA). Differences of the z-maps transferred by VMHC maps between the SA group and the HC group were detected with two-sample t-tests using the SPM12 toolkit. Multiple comparisons were conducted with Gaussian Random Field theory (When voxel-level of *P* < 0.01 and cluster-level of *P* < 0.05, the difference remains statistically significant). Receiver operating characteristic (ROC) curve method was used to identify the average value of VMHC in different brain regions of the two groups. Pearson correlation analysis was performed using Graph Pad Prism7 (GraphPad Software Inc., San Diego, CA, USA) so as to clarify the connection between the mean VMHC values of different regions in the brain and clinical features. All differences with *P* < 0.05 represents a significant difference.

## Results

### Demographics and clinical features

Seventeen SA patients (eight males and nine females) and 17 HCs (eight males and nine females) were involved in the present study. There existed no marked differences in patient age (*P* > 0.05), sex (*P* > 0.05), weight (*P* > 0.05) between the two groups. The differences in right-eye best-corrected visual acuity (*P* < 0.001) and left-eye best-corrected visual acuity (*P* < 0.05) between the two groups had statistical significance (see Table 1).

**Table 1:** Clinical characteristics of patients between SA and HC groups

	SA	HC	t-value	*p*-value
Male/female	8/9	8/9	N/A	> 0.05
Age (years)	23.56 ± 5.21	23.16 ± 5.78	−0.285	> 0.05
Weight (kg)	56.28 ± 7.37	57.85 ± 5.76	−0.489	> 0.05
Handedness	17R	17R	N/A	> 0.05
Duration (years)	18.54 ± 8.87	N/A	N/A	N/A
Best-corrected VA-Right	0.10 ± 0.05	1.05 ± 0.25	4.149	< 0.001
Best-corrected VA-Left	0.30 ± 0.09	1.00 ± 0.20	2.865	< 0.05

Notes: The independent sample t-test (*P* < 0.05 represents a significant difference) was used to evaluate differences between SA and HC groups

*Abbreviations*: *SA* Strabismus amblyopia, *HC* Healthy control, *N/A* Not Applicable, *VA* Visual acuity

### VMHC differences

**Fig. 2:**
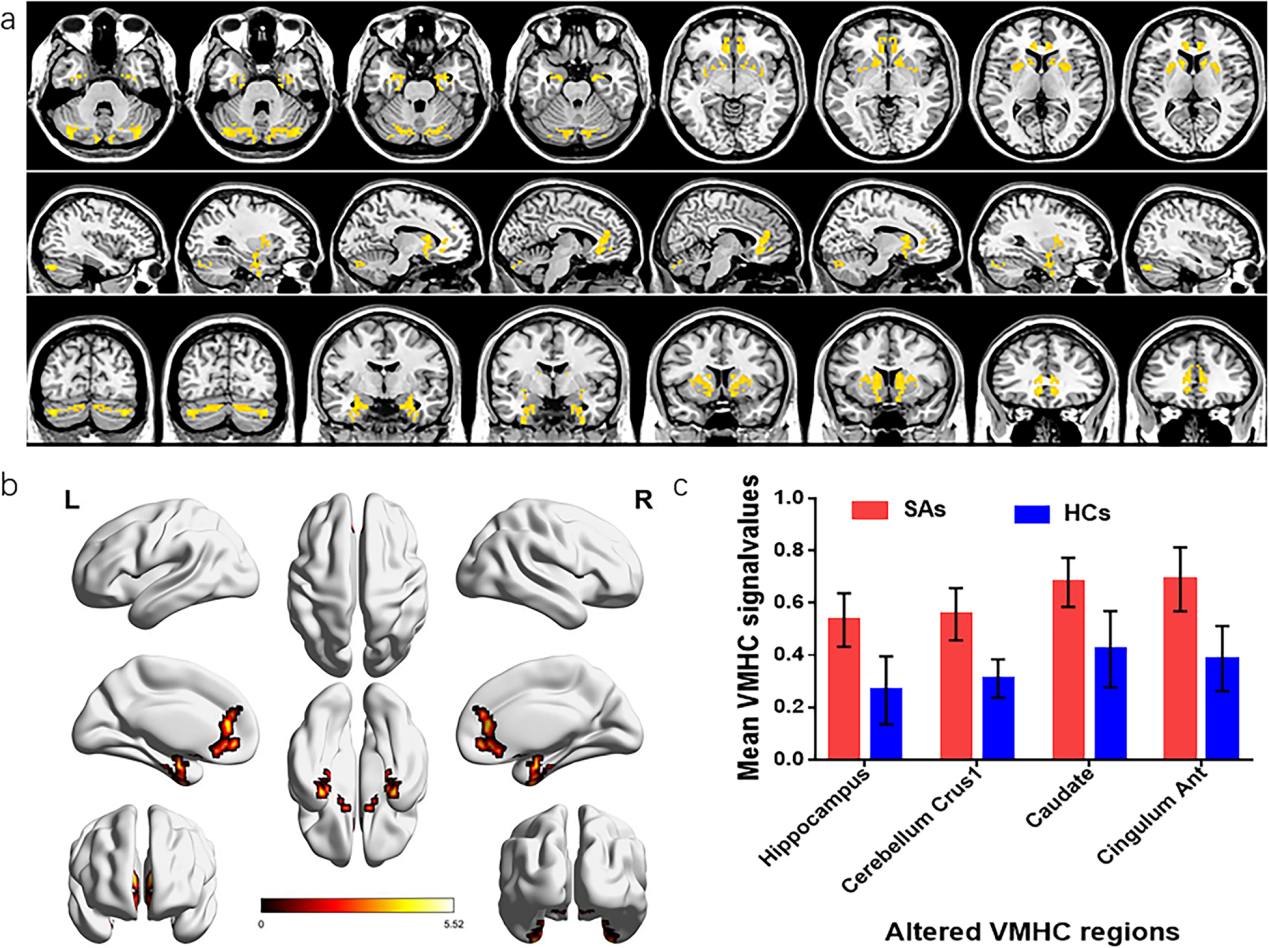
The functional connectivity of cerebral hemispheres was compared between SA and HC groups. Notes: **a** and **b**: There were significant differences in VMHC among hippocampus, cerebellar crus1, caudate nucleus and cingulate nucleus. Red or yellow indicates a higher VMHC value. The statistical threshold is set at the voxel level, and the GRF theory is used for multiple comparisons (voxel-level of *P* < 0.01, cluster-level of *P* < 0.05 and cluster≥66 voxels). **c** The average value of VMHC changed between SA and HC groups. Abbreviations: VMHC, voxel-mirrored homotopic connectivity; SA, strabismus with amblyopia; HC, healthy control; L, left and R, right

The VMHC values of bilateral caudate, cingulum ant, cerebellum crus 1, and hippocampus in patients with SA were significantly higher than those recorded in HCs (see Fig. 2a, b [yellow] or [red]; and Table 2). The differences of mean VMHC values in different brain regions between the two groups were represented in a histogram (*P* < 0.01, cluster≥66 voxels with AlphaSim being corrected) (see Fig. 2c).

**Table 2:** Brain areas with VMHC differences between SA and HC groups

VMHC	L/R	Brain regions	BA	MNI coordinates			Peak voxels	t-value
				X	Y	Z		
SA > HC								
1	R	Hippocampus	35	30	−6	−24	82	4.6
2	L	Hippocampus	35	−30	−6	−24	82	4.6
3	R	Cerebellum crus1	–	39	−78	− 33	119	5.5
4	L	Cerebellum crus1	–	− 39	−78	−33	119	5.5
5	R	Caudate	25	12	12	9	150	4.7
6	L	Caudate	25	−12	12	9	150	4.7
7	R	Cingulum ant	32	−6	33	−6	112	4.9
8	L	Cingulum ant	32	6	33	−6	112	4.9

Note: The statistical threshold is set at the voxel level, and the GRF theory is used for multiple comparisons (voxel-level of *P* < 0.01, cluster-level of *P* < 0.05 and cluster≥66 voxels)

*Abbreviations*: *VMHC* Voxel-mirrored homotopic connectivity, *BA* Brodmann area, *SA* Strabismus with amblyopia, *HC* Healthy control, *L* Left, *R* Right, *MNI* Montreal Neurological Institute, *GRF* Gaussian Random Field

### Correlation analysis

**Fig. 3:**
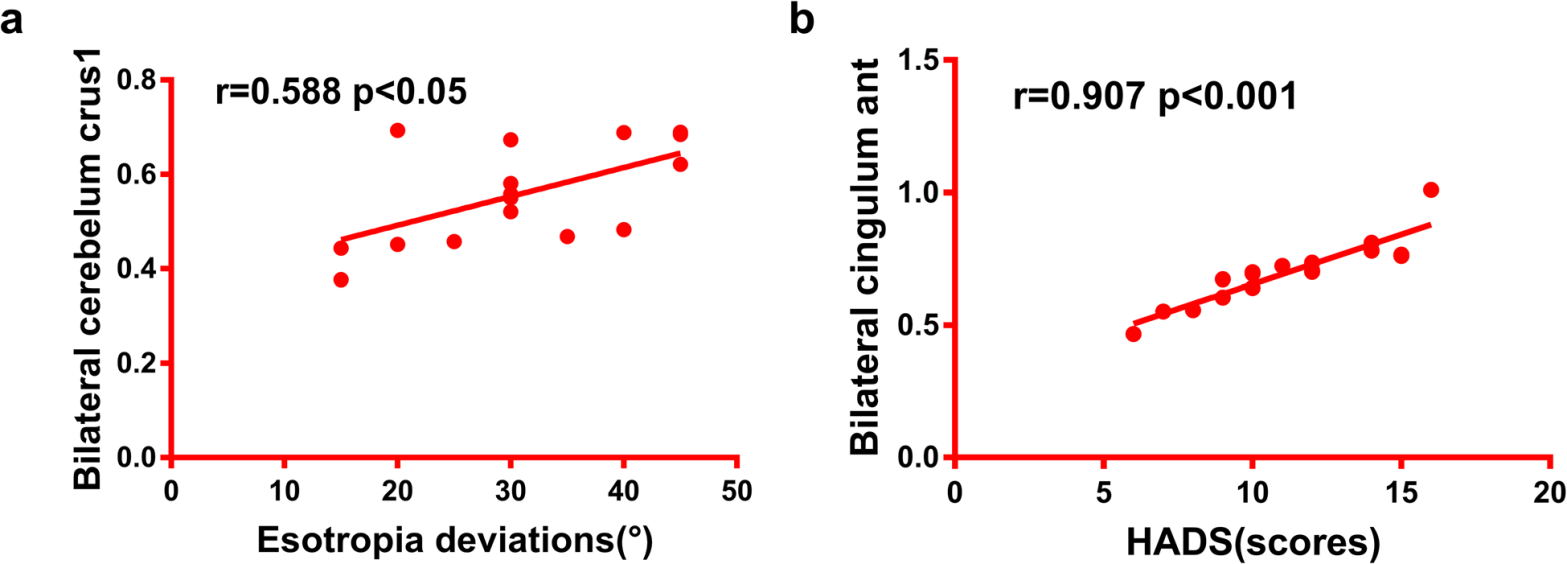
Correlations between the mean VMHC values of different regions and behavioral performance. Notes: **a** The esotropia had a positive correlation with the VMHC values of the cerebellum crus1(r = 0.588, *P* < 0.05) and **b** The HADS scale scores had a positive correlation with the VMHC values of the cingulum ant (r = 0.907, *P* < 0.001). Abbreviation: VMHC, voxel-mirrored homotopic connectivity and HADS,hospital anxiety and depression scale

In the SA group, the esotropia deviations exhibited a positive correlation with the VMHC values of the bilateral cerebellum crus 1 (r = 0.588; *P* < 0.05). There was no significant correlation between other brain regions with VMHC changes and esotropia deviations (r = − 2.43–0.254, *P* > 0.05). The hospital anxiety and depression scale (HADS) scores showed a positive correlation with the VMHC values of the cingulum ant (r = 0.907; *P* < 0.001), Additionally, there existed no significant correlation between other brain regions with VMHC changes and HADS scores (r = 0.228 ~ 0.479, *P* > 0.05).(see Fig. 3).

### ROC curve

**Fig. 4:**
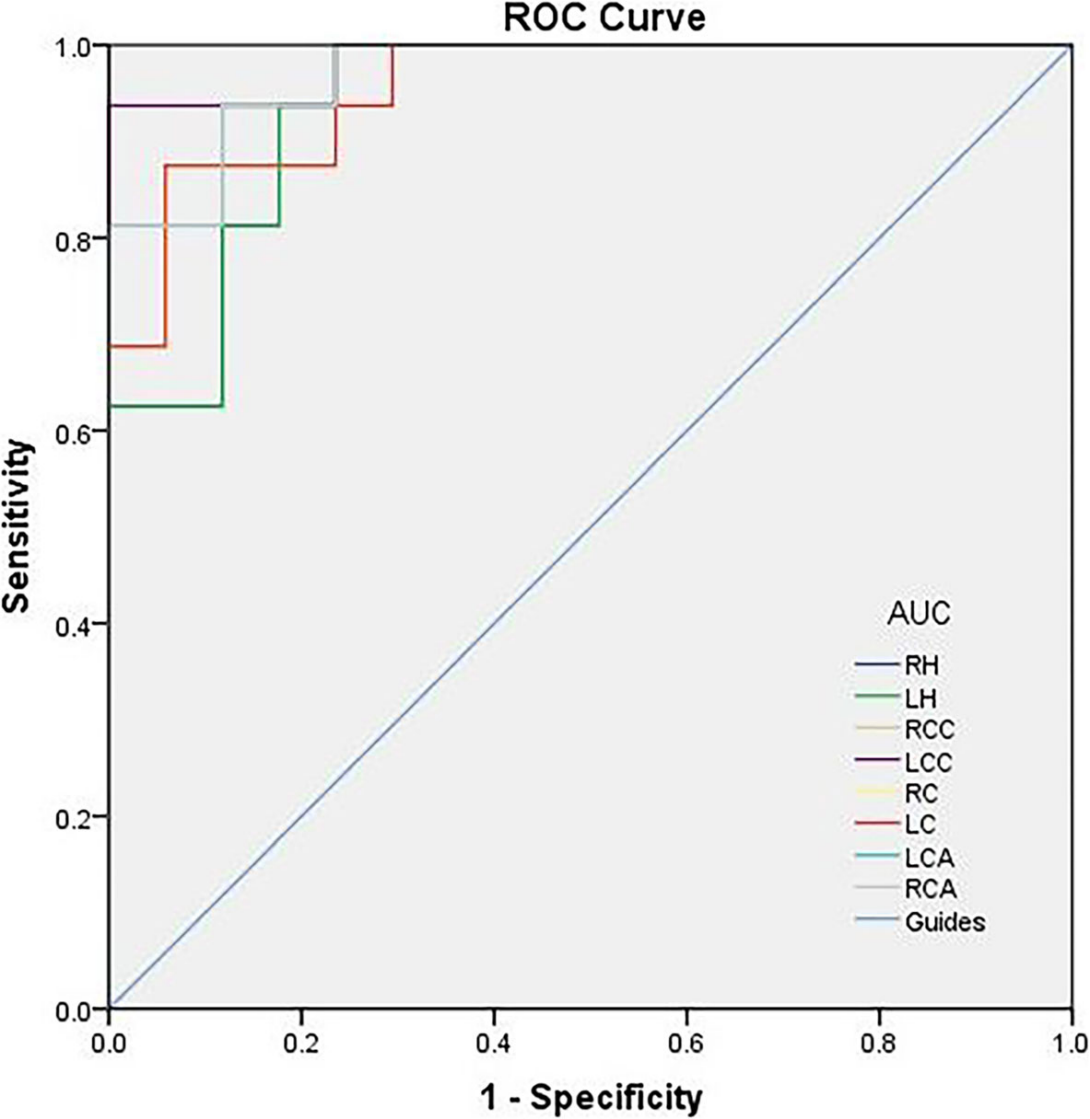
ROC curve analysis of the mean VMHC difference for altered brain regions. Notes: ROC curve: SA > HC, for the RH 0.941 (p<0.001; 95% CI: 0.867–1.000), SA > HC, for the LH 0.941 (p<0.001; 95% CI: 0.867–1.000), SA > HC, for the RCC 0.985(p<0.001; 95% CI: 0.952–1.000), SA > HC, for the LCC 0.985 (p<0.001; 95% CI: 0.952–1.000), SA > HC, for the RC 0.956 (*p* > 0.05; 95% CI: 0.895–1.000), SA > HC, for the LC (*p* > 0.05; 95% CI: 0.895–1.000), SA > HC, for the RCA 0.971 (*p* > 0.05; 95% CI: 0.924–1.000) and SA > HC, for the LCA 0.971 (*p* > 0.05; 95% CI: 0.924–1.000). Abbreviations: SA, strabismic amblyopia; HC, healthy control; ROC, receiver operating characteristic; RH, right hippocampus; LH, left hippocampus; RCC, right cerebellum crus; LCC, left cerebellum crus; RC, right caudate; LC, left caudate; RCA, right cingulum ant and LCA, left cingulum ant

We hypothesized that the VMHC differences between the SA and HC groups might be constructive diagnostic markers to distinguish the SA group from HCs. The mean VMHC values in the different brain regions were analyzed by employing the ROC curve method. The higher AUC values denote better differentiation between the two groups. The individual areas under the curve of VMHC values in different regions were presented as follows: right hippocampus (0.941; *P* < 0.001), left hippocampus (0.941; *P* < 0.001), right cerebellum crus 1 (0.985; *P* < 0.001), left cerebellum crus 1 (0.985; *P* < 0.001), right caudate (0.956; *P* > 0.05), left caudate (0.956; *P* > 0.05), right cingulum ant (0.951; *P* > 0.05), and left cingulum ant (0.951; *P* > 0.05) (see Fig. 4).

## Discussion

The SA patients had significantly increased VMHC values in bilateral cingulum ant, caudate, hippocampus, and cerebellum crus 1.The hippocampal (BA35) is an important brain structure involved in cognition. Moreover, it is a part of the limbic system of the brain, playing a certain role in working memory, specific types of monitoring targets, cognitive and emotional processing [24] as well as the function of spatial location [25]. In the study of Kana Okada [26], when the structure of the hippocampus is damaged, the escape latency of mice is prolonged during water maze training. This shows that the hippocampus has a synergistic effect on spatial reference and location. A recent monkey study [27] demonstrated that hippocampal is critical for visual memory, performing in shape recall, and image recognition. Similar studies have also showed that individuals may have recognition deficits when hippocampus is damaged [28, 29]. In the present study, we found that the VMHC value of the hippocampus was increased, which may be related to the compensatory mechanism. An electroencephalography study suggested the face-selective neuronal responses to face images diminished in amblyopic patients [30],which may cause significant cortical function deficit in face perception [31] .The cortical function is driven by both the amplitude and phase of neuronal activity. Normal people are proved to show stable homotopic resting-state functional connectivity between bilateral visual cortex [32]. Amblyopia impaired the visual acuity, image recognition and memory, which may cause the alteration of neuronal activity in interhemispheric functional connectivity of hippocampal. Admittedly, the deviation of the visual axis will cause macular not to receive efficient stimulation during the sensitive period in SA patients, The cells size connected to the nucleus layer of the lateral geniculate body connected to the amblyopia and basophilic staining of the ganglion cells are obviously reduced, while the axons of the corresponding neuro cortical cells driven by normal eyes are expanded in a complementary expansion [33]. This suggests that a similar potential compensation mechanism may promote sensory-guided motor behavior in visually impaired individuals. Increased VMHC values between the bilateral hippocampi might reflect the ability to recognize andmemorize face images in SA patients, which may reflect the ability of spatial positioning [25].

The cerebellum crus 1 is the medullary part of the cerebellum, which is associated with cognition and language execution [34, 35]. In addition, it is also related to the execution of eye movements and responsible for performing accurate eye movements [36]. Previously, it was found that the cerebellar hemisphere neuronal was activated in the spatial memory of saccade movement [37]. In a monkey study, it was found that cerebellum was involved in eye movement conjugation [38] .Min et al. [21] found that the ALFF value of the left posterior cerebellar lobe was significantly decreased in patients with SA. Lee [39] observed central positional nystagmus and spontaneous ocular volvulus in patients with lesions of the superior cerebellar feet. Shemesh [40] demonstrated that the damage to the posterior fastigial nucleus of the cerebellum decreased the accuracy of binocular saccade, and patients presented symptoms (such as dyslexia and blurred vision after changing the line of sight) and differences in binocular vision. The present study found that the esotropia showed a positive correlation with the VMHC values of the bilateral cerebellum crus 1 (r = 0.588; *P* < 0.05). We hypothesized that cerebellar damage may affect eye movement function in patients with SA. In addition, in the study conducted by Lee H [41], patients with cerebellum crus 1 diseases had symptoms such as tilt of the left head, deviation (right upper squint), and twist of the eye to the left. It can be speculated that when the brain perceives an imbalance in binocular imaging, in order to overcome this monocular blurred interference, the eye position and head position will be changed accordingly to eliminate this kind of interference, which will activate the activity of the cerebellar region and strengthen the communication and connection between the bilateral cerebellum.

The caudate (BA25) belongs to the basal nucleus and visual feedback exerts a certain role in controlling eye movement in the basal ganglia [42]. In the study of visual feedback dynamics of central neurons in cats conducted by Attila [43], it could be found that caudate nucleus neurons responded best to low spatial resolution and high time-frequency drift gratings. The spatial and temporal visual characteristics of caudate nucleus neurons offer a special visual perception function [44], which is conductive to controlling vision-guided eye movement and participating in visual movement behavior [45]. Shinya [46] observed that the tail of the caudate nucleus may have the capability of controlling visual movement, and the caudate nucleus can respond through object selection when selecting complex visual objects. In addition, Yamamoto et al. [47] also found that the neurons in the caudate nucleus have strong spatial selectivity, and electrical stimulation can easily induce spatial selective scanning. This benefits in accurately identifying the target visual object such as pilot landing [48] and billiards [49]. In a study of visual electrophysiology in cats, Nagypál [50] also found that caudate nucleus neurons may be involved in dynamic visual information processing, saccade control, and eye movement. In the current work, we observed an increase in VMHC between the hemispheres of the bilateral caudate in patients with SA, reflecting a damaged compensatory function and indicating a similar potential compensation mechanism. Therefore, we speculated that decreased saccade function and accurate location in patients with SA may activate stable homotopic connections between cortical cortices of bilateral caudate, which could be presented as altered caudate VMHC values.

The cingulum ant (BA32) has a “crescent” shape. The posterior cingulum gyrus has a visual spatial ability, which can locate its position in the visual space. Additionally, it also responds to the position of the eyeball in the orbit, the degree and direction of eye movement as well as structural visual stimulation [51]. Emma [52] found that children with optic nerve hypoplasia are often accompanied by the decreased integrity of the ventral cingulum gyrus. Besides, it is also involved in saccade movement, which is related to eye movement [53], the ALFF of the left cingulate cortex was significantly increased in patients with acute open eye injury [54]. Obviously, an increase in degree centrality in the cingulate gyrus was observed in patients with eyeball enucleation [55], suggesting that eyeball injury may cause changes in the signal values of the cingulum gyrus. Such monocular abnormal visual experiences may affect the signal transmission of nerve fibers and disturb the stable homotopic functional connectivity between bilateral visual cortex, which could be presented as increased VMHC values of bilateral cingulum gyrus. In our study, we also found that the HADS scale scores presented a positive correlation between VMHC values and the cingulum ant (r = 0.907; *P* < 0.001). The cingulum gyrus belongs to the classic circuits of the limbic system, which is closely related to memory and emotion [56],potentially indicating that the limbic system is affected in patients with SA [57]. Strabismus amblyopia influences the visual acuity, deviation of the eye position as well as eye movements and causes aesthetic problems. These problems may result in negative emotions in patients, affecting their daily psychosocial performance. In serious cases, anxiety and depression may occur [58, 59]. Consequently, we hypothesized that an increase in the VMHC signal value of the cingulum ant may reflect emotional changes in patients with SA. In our study, we speculate that the increased VMHC signal value of the cingulate gyrus is attributed to the compensatory increase in visual input defects caused by SA, which is the redistribution of brain energy consumption.

There were still some limitations in this study. Firstly, our sample size was small. In addition, we did not analyze other types of amblyopia, and have not conducted a longitudinal study on the changes of brain areas after strabismus correction. In future investigations, we will increase the sample size and include other types of amblyopia in order to further improve our understanding of this condition.

In conclusion, according to the changes in interhemispheric function and anatomical connectivity, there are abnormalities of spontaneous activity in multiple brain regions in patients with SA. These changes may provide some useful insights into the neural mechanism of SA and eye movement disorders. Moreover, the VMHC method also provides evidence for biomarkers of impaired interhemispheric connectivity in patients with SA.

## Data Availability

The datasets used or analysed during the current study are available from the corresponding author on reasonable request.
